# Successful Cognitive Aging in Rats: A Role for mGluR5 Glutamate Receptors, Homer 1 Proteins and Downstream Signaling Pathways

**DOI:** 10.1371/journal.pone.0028666

**Published:** 2012-01-06

**Authors:** Caroline Ménard, Rémi Quirion

**Affiliations:** Douglas Mental Health University Institute, Department of Psychiatry, McGill University, Montréal, Canada; National Institute on Aging Intramural Research Program, United States of America

## Abstract

Normal aging is associated with impairments in cognition, especially learning and memory. However, major individual differences are known to exist. Using the classical Morris Water Maze (MWM) task, we discriminated a population of 24-months old Long Evans aged rats in two groups - memory-impaired (AI) and memory-unimpaired (AU) in comparison with 6-months old adult animals. AI rats presented deficits in learning, reverse memory and retention. At the molecular level, an increase in metabotropic glutamate receptors 5 (mGluR5) was observed in post-synaptic densities (PSD) in the hippocampus of AU rats after training. Scaffolding Homer 1b/c proteins binding to group 1 mGluR facilitate coupling with its signaling effectors while Homer 1a reduces it. Both Homer 1a and 1b/c levels were up-regulated in the hippocampus PSD of AU animals following MWM task. Using immunohistochemistry we further demonstrated that mGluR5 as well as Homer 1b/c stainings were enhanced in the CA1 hippocampus sub-field of AU animals. In fact mGluR5 and Homer 1 isoforms were more abundant and co-localized in the hippocampal dendrites in AU rats. However, the ratio of Homer 1a/Homer 1b/c bound to mGluR5 in the PSD was four times lower for AU animals compared to AI rats. Consequently, AU animals presented higher PKCγ, ERK, p70S6K, mTOR and CREB activation. Finally the expression of immediate early gene Arc/Arg3.1 was shown to be higher in AU rats in accordance with its role in spatial memory consolidation. On the basis of these results, a model of successful cognitive aging with a critical role for mGluR5, Homer 1 proteins and downstream signalling pathways is proposed here.

## Introduction

Aging is a natural process characterized by various physical alterations: slower reaction time, cognitive alterations and modification of the muscles density [1]. However, major individual differences are observed. The concept of successful aging consists of three components namely, low probability of disease and disability, high cognitive and physical capacity and active engagement in general [1]. In the present study, we explored variability in cognitive abilities associated with normal aging using Long Evans rats as model. Similar to humans, a certain proportion of rats becomes memory-impaired with aging (AI) while others maintain spatial memory abilities comparable to young animals, and are classified as aged memory-unimpaired (AU) [2], [3], [4], [5]. Alterations in gene expression have been proposed to at least partly explain this phenomenon [6], [7]. Another hypothesis relates to the incapacity of AI subjects to adapt and properly encode new information [8]. Hippocampus-dependent spatial memory is not the only behavioural profile associated to Long Evans AI and AU sub-groups. A reduced reactivity to novelty in exploratory paradigms, gustatory/olfactory stimulus and an increased reaction to pain has also been reported in AI animals whereas in AU animals all these behaviours were similar to young rats [3].

Glutamate and its receptors are closely involved in mechanisms underlying spatial learning and hippocampus-dependent memory processes [9], [10]. The stimulation of ionotropic α-amino-3-hydroxy-5-methyl-4-isoxazole-proprionic acid (AMPA) and N-methyl-D-aspartate (NMDA) receptors induces synaptic plasticity leading to long-term potentiation (LTP) and long-term depression (LTD) [9], [11]. Alterations in synaptic plasticity, including LTP, have been previously associated with age-related memory impairments [12]. Interestingly, Lee et al. [13] had correlated NMDA receptor-independent LTD and successful cognitive aging in Long Evans rats. This form of LTD is mediated by the post-synaptic group 1 metabotropic glutamate receptors (mGluR), mGluR1α and mGluR5 [14]. mGluR1α receptors have been associated with the post-synaptic specialization of excitatory synapses and are concentrated in perisynaptic and extrasynaptic areas [15] while mGluR5 functionally interact with NMDA receptors in the postsynaptic densities (PSD) [16]. mGluR5 receptors are abundant in the adult hippocampus and cerebral cortex [17] and are involved in the maintenance of synaptic plasticity [14], [18], [19], [20], [21]. To further investigate the cellular mechanisms involved in successful cognitive aging, we studied mGluR levels and related signaling pathways in AI versus AU animals focusing on the hippocampal formation as a key structure involved in spatial learning [22].

Higher mGluR5 receptors levels were observed in AU hippocampus PSD after training compared to both AI and 6-months old animals. Metabotropic receptors closely interact with scaffolding proteins such as the family of Homers [23], [24], [25], [26]. The expression of all Homer 1 isoforms was only increased in the PSD of AU rats. Homer 1b/c are constitutively expressed and coupled with mGluR5 and its signaling effectors while Homer 1a is produced following synaptic activity and reduces the coupling of the receptor [26], [27], [28], [29]. A lower Homer 1a/Homer 1b/c ratio bound to mGluR5 receptors was observed in AU animals, leading to stronger activation of protein kinases and immediate early gene expression, especially in the CA1 hippocampal sub-field. These changes were correlated with the performance of aged rats in the MWM, which led us to propose a working model of successful cognitive aging.

## Materials and Methods

### Animals

Male Long–Evans rats were purchased from Charles River Laboratories (St Constant, QC, Canada) at 12 months of age and were housed in our animal facility until 24-months old. Another group was purchased at 3 months of age and housed in our animal facility until they were 6 months old. Animals were housed two per cage and maintained on a 12 h light/dark cycle with *ad libitum* access to food (Purina Lab Chow; Mondou, Montreal, QC, Canada) and water. Animal care, surgery and handling procedures were approved by the McGill University Animal Care Committee (protocol number 3589) and the Canadian Council for Animal Care.

### Morris Water Maze (MWM)

The long-term reference memory version of the MWM, a hippocampus-dependant behavioral task [30] was used to investigate spatial memory in Long–Evans rats. Rats were required to find, in a 1.4 m diameter pool, a submerged platform (14 cm in diameter) located 2 cm below the surface of water (24°C), rendered opaque by the addition of white paint. Animals were pseudo-randomly started from a different position on each trial and used distal visuo-spatial cues to find the hidden escape platform that remained in the centre of the same quadrant throughout training [30]. Rats were given three trials of 90 s per day for five consecutive days. Animals were guided to the platform if it was not located within 90 s. All the rats remained on the platform for 15 s before removal. After the acquisition phase on day 5, rats were given one probe trial of 60 s for which the platform was removed from the pool. The number of times the animal swim over the platform location was evaluated.

### Motor/Visual Controls

Swimming speed (see results section) and distance swam (data not shown) were used as controls of motor function, a parameter potentially altered with age. The probe test was followed by a cued trial of 60 s in which the platform was visible to assess visual deficits and motivation to escape from water.

### Classification Criteria

The learning curves and probe tests of aged rats were compared to those of 6-months old adult rats to classify them as AI or AU [3]. An animal was considered unimpaired when the learning curve and learning probe test results were less than two statistical variations to the average of adult rats. Aged rats were classified as AI when animals swam for over 25 seconds to find the platform on both days 4 and 5 (average of three trials per day). Memory impairment was confirmed with platform crossings and time spent in the target and opposite quadrants during the learning probe test. Few animals showing inconsistent results, for example latencies on days 4 and 5 similar to AI rats but the probe tests results comparable to the AU group were removed from the study and used to optimize experimental conditions.

Following the first week of training, all animals were submitted to a reverse memory paradigm to assess inhibitory learning [31]. Briefly, the platform was moved to the opposite quadrant while the position of visual cues stayed the same. Rats were then trained to find the new platform location for four consecutive days (three trials per day). A second probe test was conducted at the end of the last day (day 11). Finally, a third probe test was done seven days later to evaluate memory consolidation and retrieval. Animals were sacrificed 2–3 h after the last MWM probe test to allow immediate early gene (IEG) expression (day 18).

After each trial, rats were immediately placed under a heat lamp to dry and prevent hypothermia. To control for possible effects due to circadian cycles, all trials were performed at approximately the same time of the day between 10–15 h. Data derived from the MWM task were recorded on computer using a video tracking system (HVS, Buckingham, UK).

### Antibodies

mGluR5 antibody was procured from Millipore (Billerica, MA, USA) and used at a 1/1000 dilution for Western Blot. Homer 1 (1/200) and actin (1/2000) antibodies were purchased from Santa Cruz Biotechnology (Santa Cruz, CA, USA). The PSD95, p42/44 (Thr202/Tyr204), p42/p44, p70S6K (Thr389), p70S6K (Thr412/Ser424), p70S6K, mTOR (Ser2448), mTOR, CREB (Ser133) and CREB antibodies were all used at a 1/1000 dilution for Western Blot and purchased from Cell Signaling (Danvers, MA, USA). All other antibodies were procured from Abcam (Cambridge, MA, USA) and also used at a 1/1000 dilution. For immunohistochemistry experiments, mGluR5 and Homer 1b/c antibodies were incubated at 1/1000 and 1/200 dilution, respectively. For immunofluorescence co-localization, mGluR5, Homer 1a, Homer 1b/c, PKCγ (Thr674) and mTOR (Ser2448) were incubated at 1/100 dilution while Arc and MAP2 were used at 1/250 dilution.

Specificity of mGluR5 and Homer 1 antibodies has been established using Western Blot and immunohistochemistry experiments. As shown in the supplemental figure S1A, a band around the expected molecular weight (132 kDa) is strongly reactive to mGluR5 antibody. Incubation of this antibody with the mGluR5 peptide (provided by the company) completely blocked the interaction in both Western Blot and immunohistochemistry analyses. Specificity of the anti-rabbit secondary antibody was verified as well with the omission of the primary antibody. Incubation of the tissue preparation with Homer 1a primary antibody produced non-specific bands (Fig. S1B). Preadsorption with Homer 1a peptide blocked the interaction of only one band (mol. weight between 30 and 36 kDa), which was close to the expected molecular weight (30 kDa). Again, the specificity of the anti-goat secondary antibody was verified with omission of the primary antibody. Homer 1b/c primary antibody was highly specific with only one reactive band around 45 kDa (Fig. S1C). Incubation with the peptide completely blocked the interaction in both Western Blot and immunohistochemistry.

### Subcellular fractionation and PSD isolation

Animals were sacrificed by decapitation and brains were dissected to collect the hippocampal formation and adjacent cortex and the tissue was quickly frozen in isopentane at −80°C. The preparation included the hippocampus, entorhinal, perirhinal and portions of adjacent neocortices [32]. To compare protein levels, tissues were collected in 7 ml of Tris-acetate buffer (50 mM, pH 7.4) containing 100 µM EGTA, 0.32 M sucrose and several protease inhibitors (leupeptin 5 µM, phenylmethylsulfonyl fluoride 200 µM, N-tosyl-L-phenylalanine chloromethyl ketone 1 µg/ml, Sigma-Aldrich Canada, Oakville, Ont.). Subsequently, samples were homogenized with a polytron and total protein concentrations were determined using bicinchoninic acid protein assay kit (Pierce, Rockford, IL, USA). Two ml of each homogenate sample was frozen for Western Blot and immunoprecipitation experiments.

To obtain membrane fractions, 5 ml of sample homogenates were purified by differential centrifugation as reported previously [33]. Briefly, homogenates were centrifuged at 1,000×g for 10 min. Supernatants were then centrifuged at 14,000×g for 20 min and the resulting pellet was defined as the crude synaptosomal fraction. Synaptosomes were resuspended in Tris-acetate buffer (50 mM, pH 7.4, 100 µM EGTA, 0.32 M sucrose and proteases inhibitors).

To purify the PSD, the synaptosomal fraction was diluted with 20 mM Tris-HCl, pH 6.0, 0.1 mM CaCl_2_ containing 1% Triton X-100, mixed for 20 min at 4°C with agitation and then centrifuged at 40,000×g for 20 min at 4°C [34]. The pellet containing the isolated synaptic junctions was collected. To separate presynaptic components from the PSD, the pellet was resuspended in 20 mM Tris-HCl, pH 8.0, 0.1 mM CaCl_2_ containing 1% Triton X-100. Samples were mixed again for 20 min at 4°C with agitation and then centrifuged at 40,000×g for 20 min at 4°C. The insoluble pellet containing the PSD fraction was suspended in Tris-acetate buffer (50 mM, pH 7.4, 100 µM EGTA, 0.32 M sucrose and proteases inhibitors) and stored at −80°C until use. Purification of the PSD was controlled by Western Blot using PSD95 to assess the protein enrichment (Fig. S1D).

### Electrophoresis and immunoblotting

Western blot analysis was carried out on aliquots of homogenates and PSD fractions obtained from AI, AU and 6-months old adult rat brains. Aliquots were subjected to sodium dodecyl sulfate polyacrylamide gel electrophoresis (SDS-PAGE) on denaturing NuPAGE® Novex 4–20% Bis–Tris gel (Invitrogen, Carlsbad, CA, USA). Proteins were transferred onto Hybond-C nitrocellulose membranes (Amersham Biosciences, Little Chalfont, UK). To block nonspecific sites, membranes were first incubated for 1 h at room temperature in phosphate-buffered saline (PBS) containing 2% bovine serum albumin (BSA). Membranes were next incubated with primary antibodies directed against group 1 mGluR (mGluR1α and mGluR5), Homer proteins (Homer 1a, Homer 1b/c, Homer 2, Homer 3) and signaling pathways enzymes - PKCγ (Thr674), PKCγ, p42/44 MAPK (Thr202/Tyr204), p42/44 MAPK, p70S6K (Thr389), p70S6K (Thr421/Ser424), p70S6K, mTOR (Ser2448), mTOR, CREB (Ser133), CREB - in PBS containing 2% BSA. Bands corresponding to proteins were detected with peroxidase-conjugated secondary antibody (Santa Cruz Biotechnology, CA, USA) and Western Lightning Chemiluminescence Reagent Plus (Perkin Elmer, Boston, MA, USA) on Kodak BioMax MS film (Amersham Biosciences). The same labelling procedure was used for negative controls but primary antibody was omitted. Actin level was used as a loading control. Immunoblots were placed on a Northern light illuminator and computer-generated images were analyzed semiquantitatively by densitometry with a microcomputer imaging device (Imaging Research, MCID, St. Catharines, ON, Canada).

### Immunohistochemistry

Coronal sections (20-µm) of perfused brains (fixed with 4% paraformaldehyde and cryoprotected in 30% sucrose solution) at the level of dorsal hippocampus were washed in PBS (pH 7.4) for 5 min then immersed in methanol containing 0.3% H_2_O_2_ for 30 min at room temperature to quench endogenous peroxidases. After three washes of 5 min each in PBS, sections were preincubated with 10% normal goat serum (NGS) at room temperature for 1 h, followed by incubation with polyclonal mGluR5 or Homer 1b/c primary antibodies (1/1000 or 1/200 in PBS with 1% NGS) overnight at 4°C. Subsequently, sections were washed 3 times in PBS and incubated in biotinylated anti-rabbit IgG (1∶200, Vector Laboratories, Burlingame, CA, USA) in 1% NGS for 30 min. After washing in PBS, sections were finally incubated with avidin biotinylated enzyme complex (ABC reagent, Vector Laboratories, Burlington, ON, Canada) diluted in PBS for 30 min. The peroxidase reaction was carried out with 0.02% hydrogen peroxide and 3,3′-diaminobenzidine tetrahydrochloride (0.1% in 100 mM Tris-HCl buffer, pH 7.4). Sections were then washed in tap water, cleaned and mounted on Superfrost Plus slides (Thermo Scientific, Portsmouth, NH, USA). Immunohistochemical staining was visualized using bright-field microscopy at 2× and 10× magnification. Controls were prepared using the same labelling procedure, but primary antibody was omitted (Fig. S1).

### Immunofluorescence

Coronal 20-µm sections of perfused brains at the level of the dorsal hippocampus were washed in PBS (pH 7.4) for 5 min. Sections were permeabilized with 0.2% Tween 20 in PBS for 10 min at room temperature then processed for immunofluorescence labelling. In brief, sections were first incubated in 10% NGS (or normal horse serum for Homer 1a) diluted in 0.1 M PBS with 0.05% Tween 20 (PBST) and 1% BSA for 60 min at room temperature, followed by overnight incubation with primary antibodies at 4°C in a solution of 1% serum and 1% BSA in 0.1 M PBST. After 3 washes in PBS, sections were incubated with corresponding secondary antibodies (1∶500, Invitrogen, Carlsbad, CA, USA) conjugated with Alexa Fluor 488 or Alexa Fluor 568 in 1% BSA in PBST for 2 h at room temperature in dark. Sections were washed three times with PBS for 5 min each in dark. Nuclei were stained with Hoechst solution (2 µg/ml, Invitrogen, Carlsbad, CA, USA) for 5 min and subsequently the sections were washed and coverslipped with Fluoromount-G (Southern Biotech, Birmingham, AL, USA). Pictures were taken at 40× magnification with an Axio Observer microscope with Apotome (Carl Zeiss MicroImaging GmbH, Germany).

### Immunoprecipitation

20 µg of PSD and synaptic membrane proteins were immunoprecipitated to investigate mGluR1α/5-Homer protein interactions. Samples were suspended in 150 µl of lysis buffer (100 mM Tris/HCl, 100 µM EGTA, pH 7.4) containing 1% SDS and heated at 100°C for 5 min, followed by dilution in 600 µl of cold lysis buffer containing 2% Triton X-100. Insoluble materials were removed by centrifugation at 15,000×g for 30 min. Protein A-agarose beads (Millipore, Billerica, MA, USA) and antibody (3 µl) mixtures were added to the supernatant and incubated overnight with agitation at 4°C. Immunoreactive complexes were recovered by brief centrifugation and pellets were washed with lysis buffer-2% Triton X-100. Proteins were finally eluted in 2× Laemmli sample buffer containing 4% SDS, 20% glycerol, 10% 2-mercaptoethanol, 0.004% bromophenol blue and 0.125 M Tris HCl, pH 6.8. (Sigma-Aldrich Canada, Oakville, Ontario) and heated at 100°C for 10 min. Immunoprecipitated proteins were subjected to SDS-PAGE and immunoblotting (see preceding section).

### Statistical analysis

In all cases, data are presented as means ± SEM. MWM latencies and platform crossings were analyzed by two-way ANOVA followed by Bonferroni *post hoc* analysis with GraphPad Prism software. Swim speed, time spent in target and opposite quadrants and protein levels were analyzed by one-way ANOVA followed by Bonferroni *post hoc* analysis. Behavioural data correlations were evaluated with a two-tailed Pearson's test. Significance level was set at *p*<0.05.

## Results

### Alterations in cognitive abilities with normal aging

**Figure 1:**
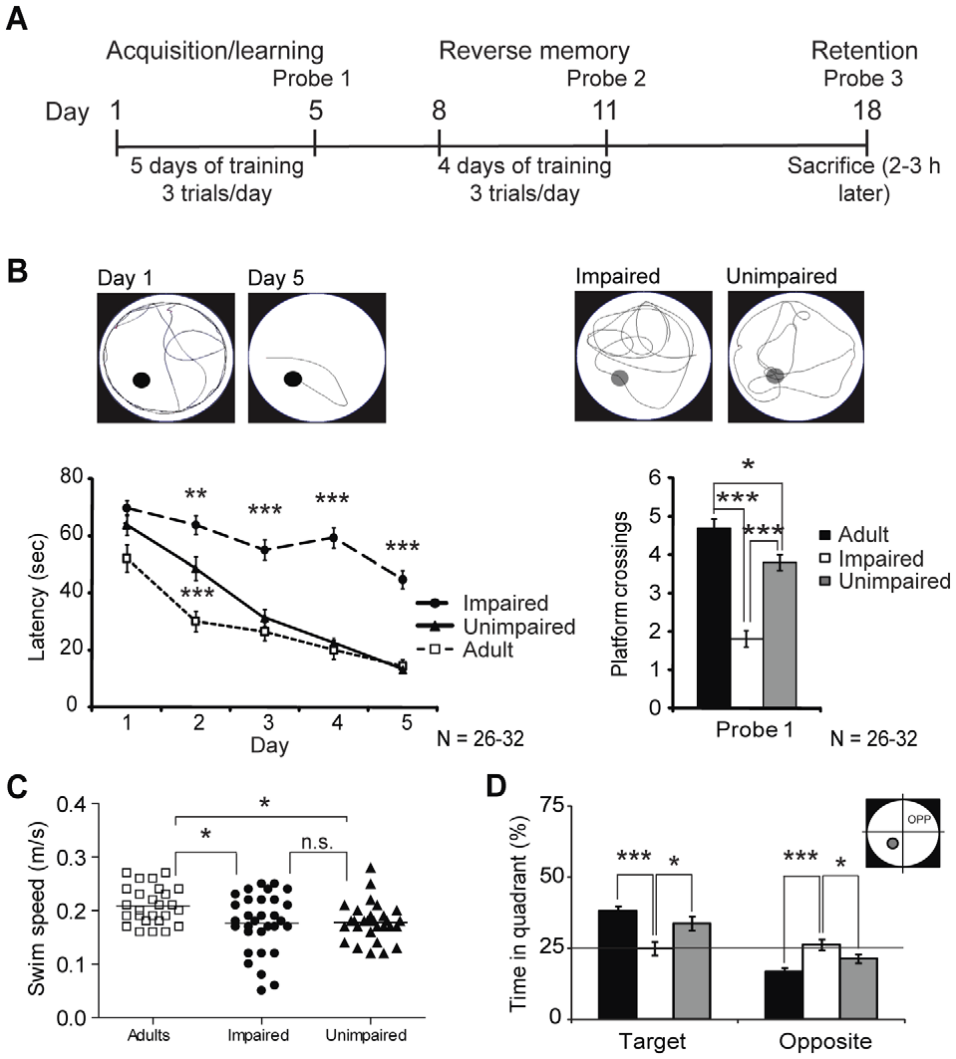
Effect of aging on the MWM acquisition task. A) Behavioral protocol schedule. B) Learning curves of the AI (Impaired), AU (Unimpaired) and 6 months old (Adult) rats for week 1 as well as average probe 1 test results (platform crossings) for all groups. Representative tracking pictures for day 1, day 5 and AI vs AU probe tests are presented. C) Swim speed distribution in all groups during probe 1 test. D) Time spent in target and opposite quadrants during learning of probe test. Data represent mean ± SEM for all animals of each group (N = 26–32), *p<0.05, **p<0.01, ***p<0.001.

The effect of normal aging on spatial memory was evaluated on the basis of MWM performance in 24-months old rats. We compared learning curves of aged rats with those of young animals (6-months old). The behavioural training protocol is presented in Figure 1A. As shown in Fig. 1B, the average learning curve of the AI (N = 31) animals was significantly different from the AU (N = 32) and adult rats (N = 26) for the last 4 days (**p<0.01, ***p<0.001). A considerable difference was also observed between the performance of adult and AU rat on day 2 (***p<0.001). The average performance of the AI animals was much lower compared to the AU and adult rats for the first probe test (*** *p*<0.001). The adult animals also crossed the platform more often than the AU rats (*p<0.05), which is probably due to a slightly faster swimming speed (*p<0.05) for these animals (Fig. 1C). Motor activity as shown by the swim speed was comparable for both groups of aged rats (Fig. 1C, 0.167±0.009 m/s for AI vs 0.169±0.006 m/s for AU). Time spent in the target and opposite quadrants was evaluated to confirm memory status. As depicted in Fig. 1D, adult and AU rats spent significantly more time (38.06±1.83%, ***p<0.001 and 33.76±2.34%, *p<0.05 respectively) in the platform quadrant (target) while AI animals swam randomly during the learning probe test; 24.90±2.23% of time in the target quadrant versus 26.12±1.84% in the opposite quadrant.

**Figure 2:**
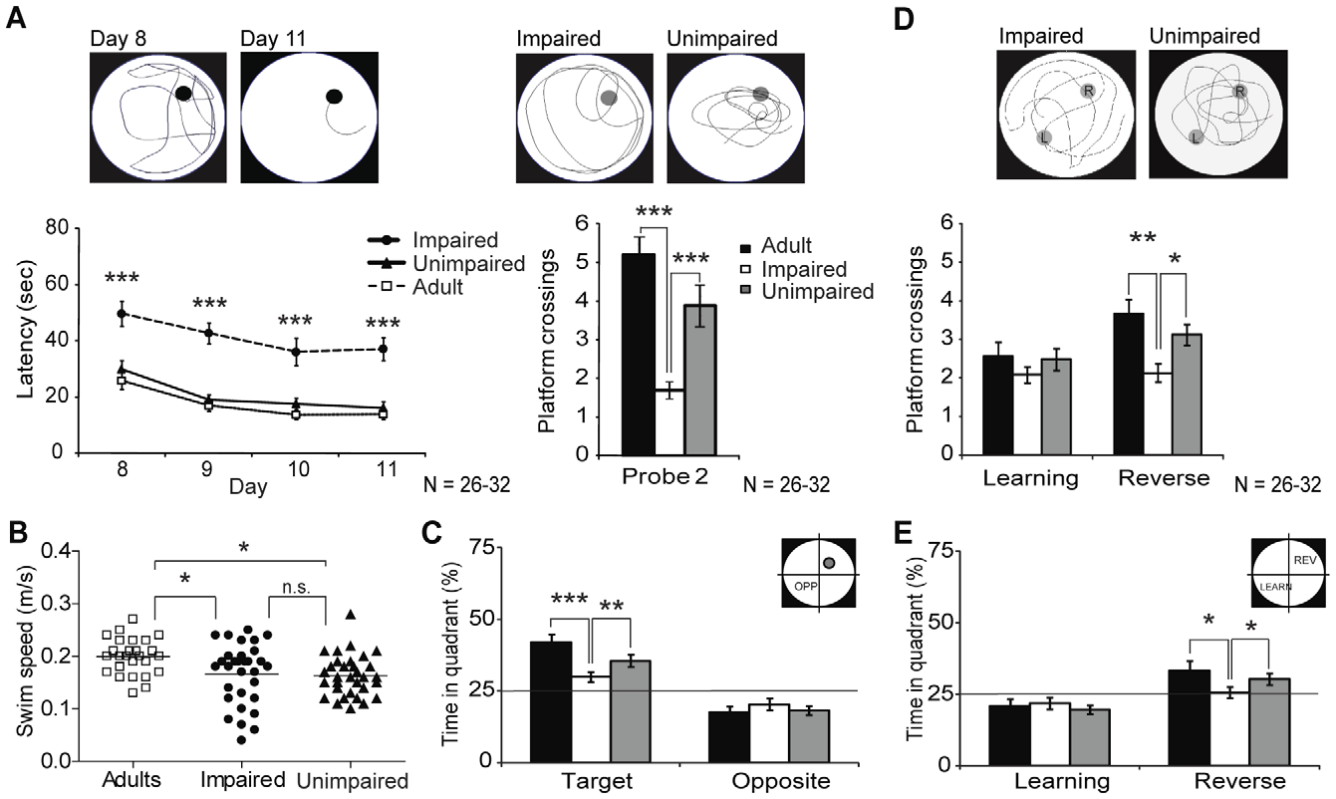
Effect of aging on reverse memory and retention. A) Learning curves and representative tracking pictures for week 2 as well as average results for the reverse probe test on day 11 (probe 2). B) Swim speed distribution in all groups during probe 2 test. C) Time spent in target and opposite quadrants during the reverse probe test. D) Results of the retention probe test conducted one week after the reverse probe test. Representative tracking pictures for AI and AU groups are presented. E) Time spent in learning and reverse platform quadrants during the retention probe test. Data represent mean ± SEM for all animals of each group (N = 26–32), *p<0.05, **p<0.01, ***p<0.001.

Aging has been shown to alter synaptic plasticity associated with new memories [8]. This hypothesis was evaluated using a reverse memory task. The platform position was modified and rats were trained again, 3 times a day, for 4 consecutive days. As shown in Figure 2A, the average learning curve of AU rats overlapped with that of adult animals while the performance of AI animals was getting worse (***p<0.001). A second probe test was conducted on day 11 and similar to the first test, AI animals crossed the platform fewer times (Fig. 2A ***p<0.001). No significant change was observed for young adult versus AU animals. Motor activity was evaluated by swim speed and results were identical to the first probe test with adult rat group swimming slightly faster (Fig. 2B, *p<0.05). Time spent in the new target and opposite quadrants was assessed to evaluate if aged animals were going back to the initial platform position (i.e. learning), suggesting an impairment of new memories formation. Interestingly, adult and AU animals spent similar percentage of time in the new target quadrant (Fig. 2C, 39.36±1.75 vs 35.42±2.32% respectively, *p<0.05). Similar to the first learning probe test, AI rats spent 25% of the time swimming in both target and opposite quadrants. Finally, an evaluation of memory consolidation was done with a third probe test one week later (probe 3). The AU animals again crossed the previous platform position (reverse) as often as the adult rats while the AI rats struggled to remember it (Fig. 2D, **p<0.01, *p<0.05). None of the groups went back to the learning platform quadrant significantly (Fig. 2E); adult and AU rats were focused on the reverse platform quadrant (*p<0.05) while AI animals still swam randomly (25.57±1.80%).

**Figure 3:**
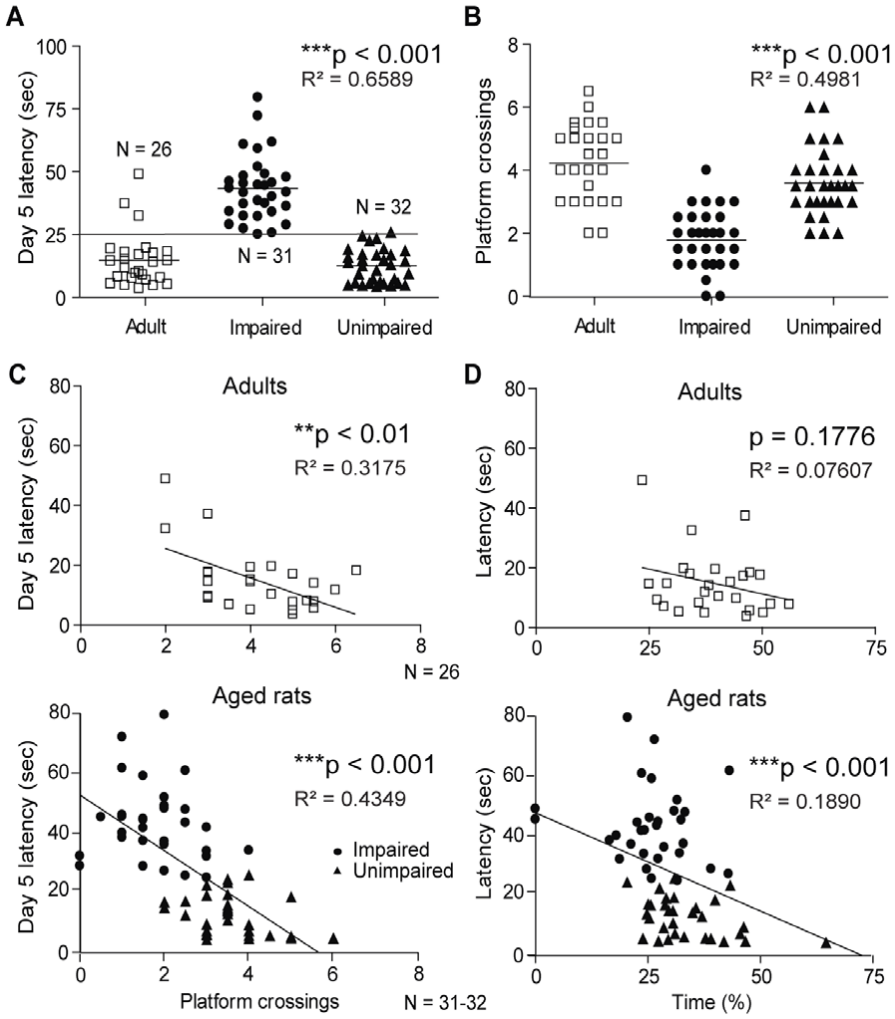
Correlation between day 5 latency and cognitive abilities. A) Distribution of day 5 latency for all 3 groups; this parameter is used to correlate protein levels with MWM performance. B) Number of platform crossings distribution during learning and reverse probe tests (mean of probes 1 and 2) for all groups. C) Day 5 latency correlation with the mean of learning and reverse probe test results for both adult and aged rats. D) Correlation of day 5 latency with the mean of time spent in target quadrant during learning and reverse probe tests for both adult and aged rats. Data represent mean ± SEM for all animals of each group (N = 26–32), **p<0.01, ***p<0.001.

As mentioned in the Materials and Methods section, the main criteria to classify the aged rats in either impaired or unimpaired sub-groups was day 5 latency. Individual performance of aged rats was compared to the mean value of adult animals and as shown in Fig. 3A, the criterion was set at 25 seconds (***p<0.001). Learning and reverse probe test results were also taken into account for classification. The distribution cleavage is less evident but still significantly different among the groups (Fig. 3B, ***p<0.001). Correlation between day 5 latency and average probe test performance for each individual was determined to confirm the criterion validity. As presented in Fig. 3C, day 5 latencies were correlated with the mean of learning and reverse probe tests for adult animals (**p<0.01), with correlation being stronger for aged rats (Fig. 3C, ***p<0.001). Finally, correlation between day 5 latencies and time spent in overall target quadrant was evaluated to confirm memory adaptative abilities. While adult animal performances show a tendency towards adaptative abilities, correlation for aged rats was also strongly significant (Fig. 3D, ***p<0.001), confirming that the 24-months old animals spending more time swimming in the target quadrant during learning and reverse probe tests found the platform faster on day 5 as well.

### Decrease in metabotropic glutamate receptor levels in aged memory-impaired animals

**Figure 4:**
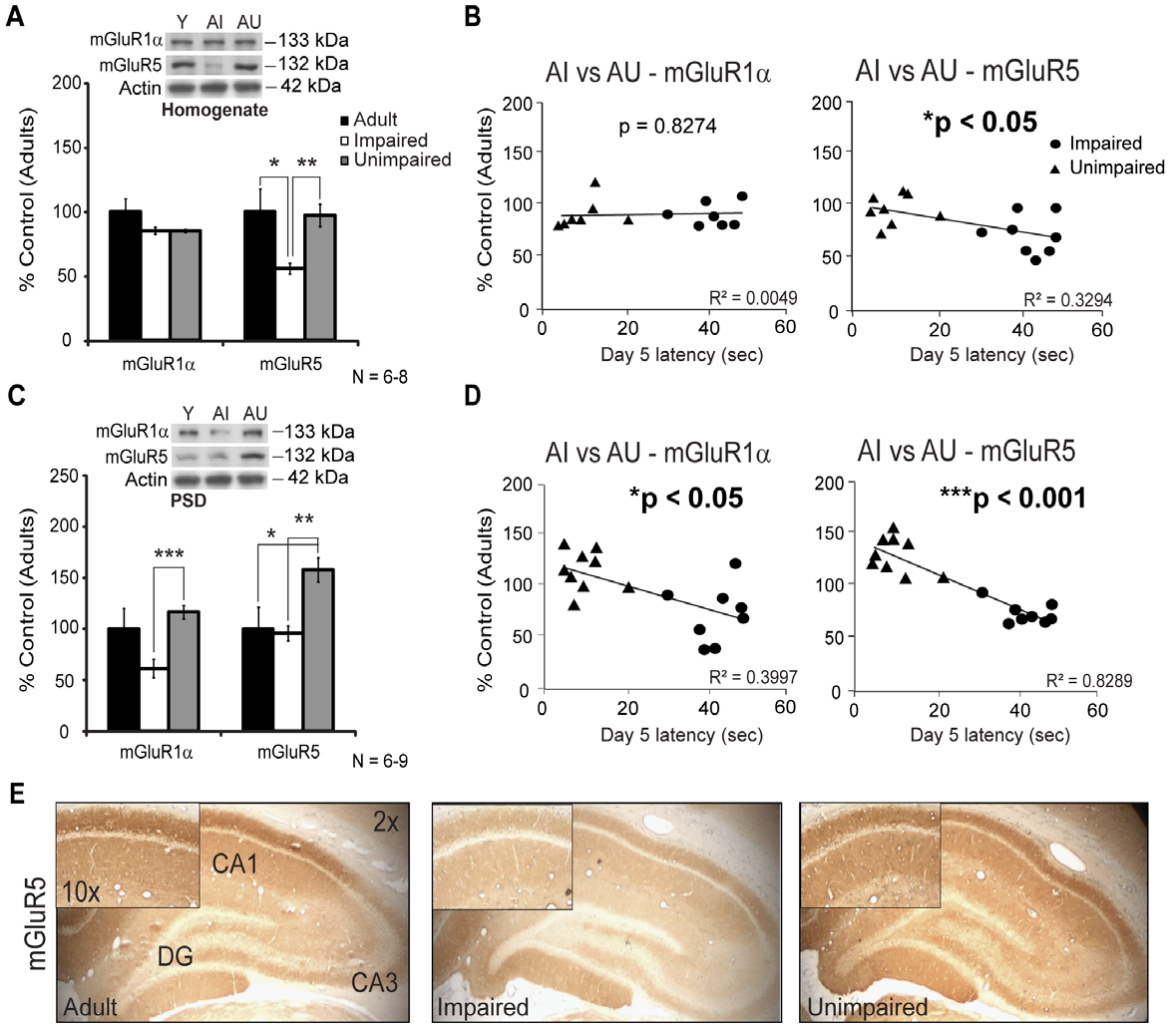
Comparison of group 1 mGluR levels in adult (Y), AI and AU hippocampal formations. A) mGluR5 protein level was decreased in the hippocampal homogenates of AI animals and B) this modification was correlated to the MWM performance for aged rats. C) mGluR1α receptor level in the PSD was decreased for AI rats while AU animals showed an increase in mGluR5 level. D) Both alterations were correlated to the MWM day 5 latencies for aged rats. E) mGluR5 immunostaining was strongly reduced in the AI hippocampus particularly in the dendrites of the CA1 region. AU displayed small increase in mGluR5 level in CA1 and DG. Western Blot values represent mean ± SEM of three separate experiments (N = 6–9) and the data are expressed as percentage of control (6-months adult rats), *p<0.05, **p<0.01, ***p<0.001.

Considering the importance of metabotropic glutamate receptors LTD for successful aging and cognition [13], we investigated group 1 mGluR receptor expression in the adult, AI and AU hippocampal formation following MWM training to explain the performance discrepancies. The mGluR1α protein level was unaltered in the homogenates of all groups (Fig. 4A). However, mGluR5 was decreased in the AI homogenate samples (Fig. 4A, 56%±4% vs 97%±9% AI vs AU, **p<0.01 and 100%±17% AI vs adult rats, *p<0.05). As presented in Fig. 3, day 5 latency is a good indicator of cognitive faculties. Thus, the correlation between this parameter and molecular changes was evaluated for each individual animal. Protein level of mGluR5 correlates with aged rats MWM performance on day five (Fig. 4B, *p<0.05). Since synaptic transmission is concentrated at synapses [9], PSD fraction was purified and mGluRs expression level was evaluated. Receptor level of mGluR1α was reduced in the PSD of AI animals (61%±9%, ***p<0.001, Fig. 4C), while the level of mGluR5 receptor was significantly increased in the PSD of the AU group (Fig. 4C, 158%±12%), not only compared to AI animals (96%±7%, **p<0.01) but also with young adults (100%±21%, *p<0.05). The correlation between the behavioural data and mGluR1α in aged rats was significant (Fig. 4D, *p<0.05) but was stronger for mGluR5 receptor (***p<0.001). Changes in mGluR5 receptor levels were restricted to a specific area of the hippocampal formation. Indeed, mGluR5 staining was predominant in the dendrites of the CA1 sub-field and reduced in the AI group (Fig. 4E).

### mGluRs and Homer proteins interactions are altered in the hippocampal formation of aged memory-impaired animal

**Figure 5:**
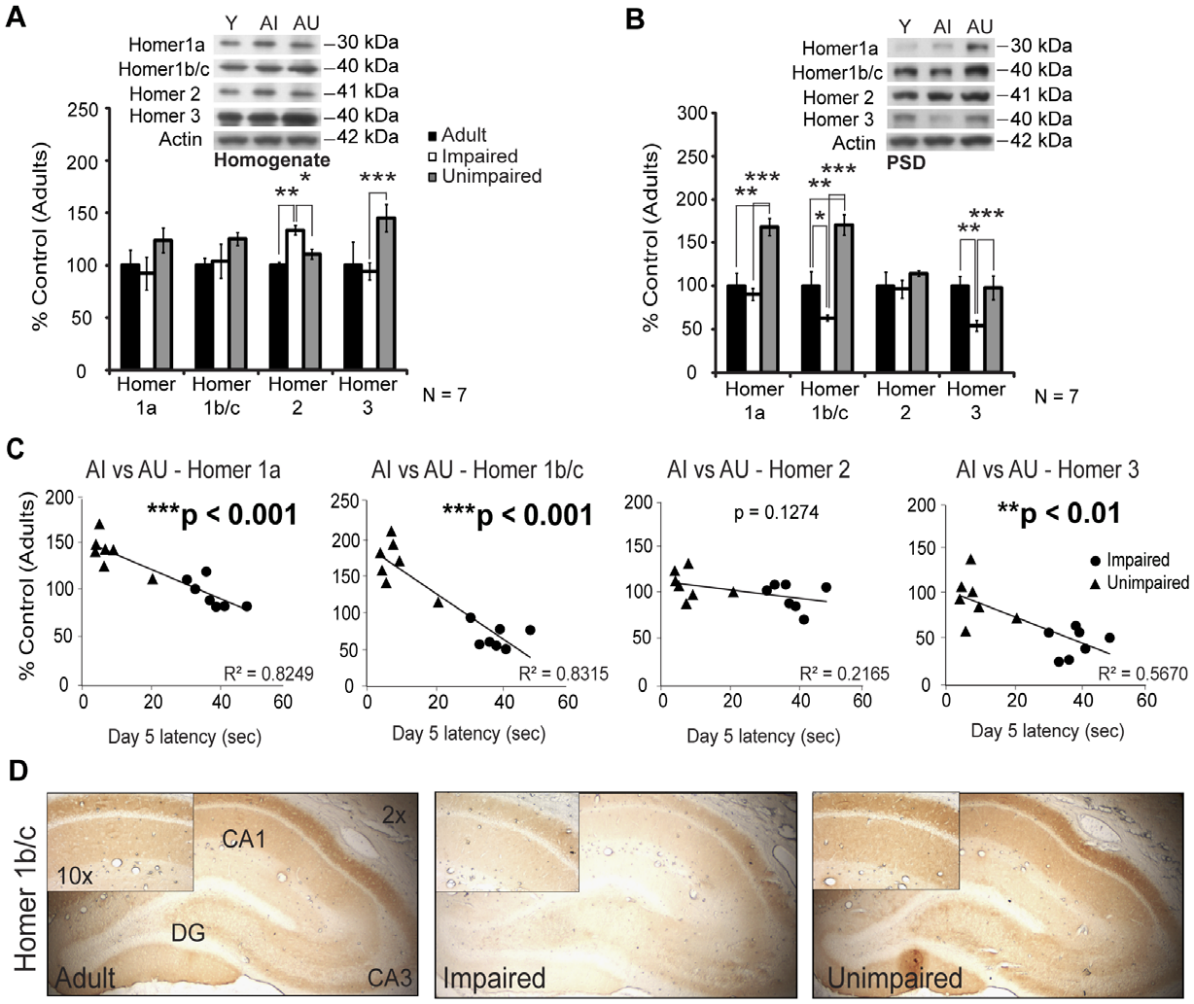
Comparison of Homer protein levels in adult (Y), AI and AU hippocampal formations. A) Homer 3 protein level was increased in AU homogenates, while Homer 2 level was increased in AI hippocampal formation. B) Homer 1a and 1 b/c protein levels were considerably increased in AU PSD. The AI animals displayed reduced levels of Homer 3 in the PSD. C) All these alterations in the PSD were correlated to MWM day 5 latencies. D) Similar to mGluR5, Homer 1 b/c immunostaining was reduced in the AI hippocampus, mainly in the CA1 area. Western Blot values represent mean ± SEM of three separate experiments (N = 7) and the data are expressed as percentage of control (6-months adult rats), *p<0.05, **p<0.01, ***p<0.001.

Group 1 metabotropic glutamate receptors are associated with Homer scaffolding proteins [23], [24], [25], [26]. The Homer 1b/c, Homer 2 and Homer 3 long isoforms are constitutively expressed and mediate mGluR signaling. An increase in synaptic activity induces the transcription of the dominant negative Homer 1a short isoform [26], [27], [28], [29]. A significant up-regulation in the level of Homer 3 protein was observed in hippocampal homogenates of the AU compared to AI animals (Fig. 5A, 145%±13% vs 94%±8%, ***p<0.001) while AI animals demonstrated an increase in Homer 2 (134%±5%, *p<0.05, **p<0.01). The AU animals demonstrated strong increase in the levels of Homer 1a (Fig. 5B, 168%±10%, **p<0.01, ***p<0.001) and Homer 1b/c (171%±12%, **p<0.01, ***p<0.001) protein in the PSD compared to both AI and young adult rats, while Homer 3 level was decreased in the AI group (54%±6%, **p<0.01, ***p<0.001). MWM performance were significantly correlated with protein levels of Homer 1a (Fig. 5C, ***p<0.001), Homer 1 b/c (***p<0.001) and Homer 3 (**p<0.01) in the PSD. Similar to mGluR5, Homer 1 b/c immunostaining was more intense in dendrites of the CA1 hippocampal sub-field particularly in AU animals (Fig. 5D).

**Figure 6:**
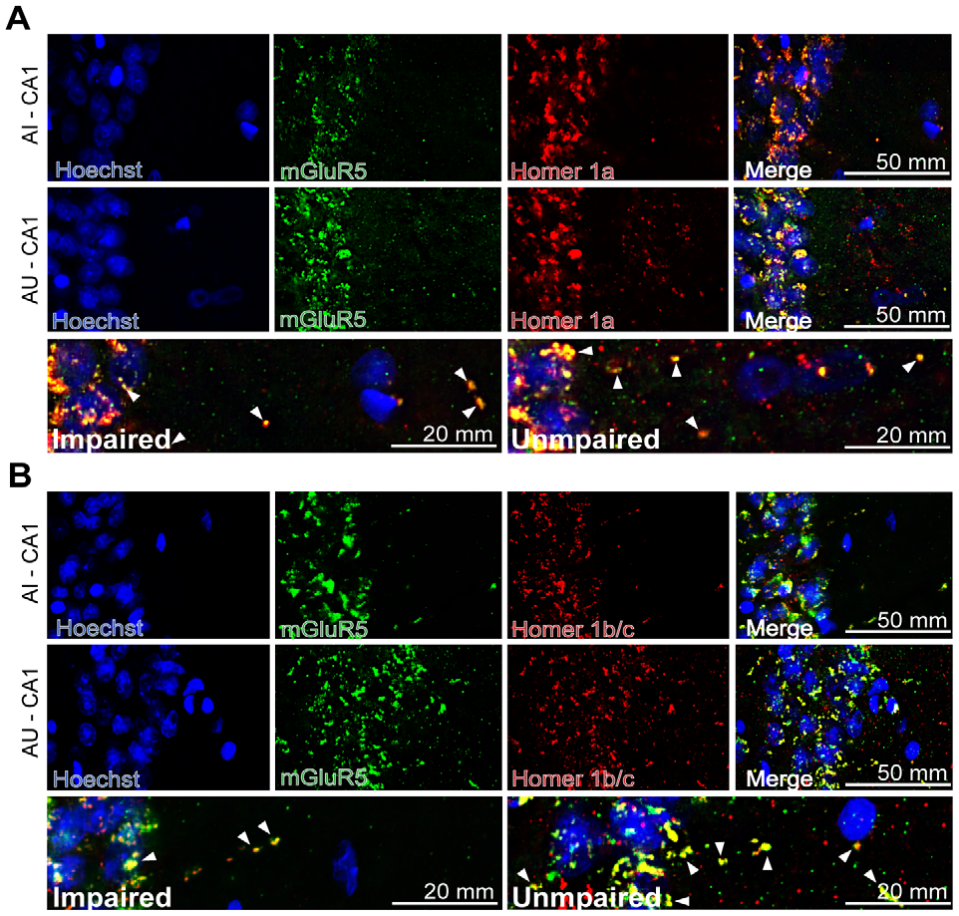
Co-localization of mGluR5 and Homer 1 isoforms. A) Homer 1a immunofluorescence and co-localization with mGluR5 was overall stronger in the cell bodies and dendrites of CA1 area in AU animals. At higher magnification (bottom), both mGluR5 and Homer 1a appear to be expressed more in the dendrites of AU animals but the co-localization seems to be similar for both AI and AU rats. B) mGluR5 and Homer 1 b/c co-localization was also overall higher in the cell bodies and dendrites of the CA1 region in AU animals. Both mGluR5 and Homer 1b/c are more expressed and co-localized in the AU dendrites compared to AI (bottom).

In addition, we investigated the regional specificity of the expression of mGluR5 and Homer 1 isoforms using double immunofluorescence. As Homer 1a is an IEG translated following synaptic activity [35], it is mainly co-localized with mGluR5 in the nuclear area of the cells (Fig. 6A). IEG expression was expected since the animals were sacrificed 2–3 hours after the last probe test. Homer 1a expression was more abundant in CA1 neurons of AU animals and occasionally co-localized with mGluR5 in dendrites (Fig. 6A). Homer 1 b/c expression was strongly co-localized with mGluR5 in CA1 neurons and especially in dendrites, particularly in AU rats (Fig. 6B).

**Figure 7:**
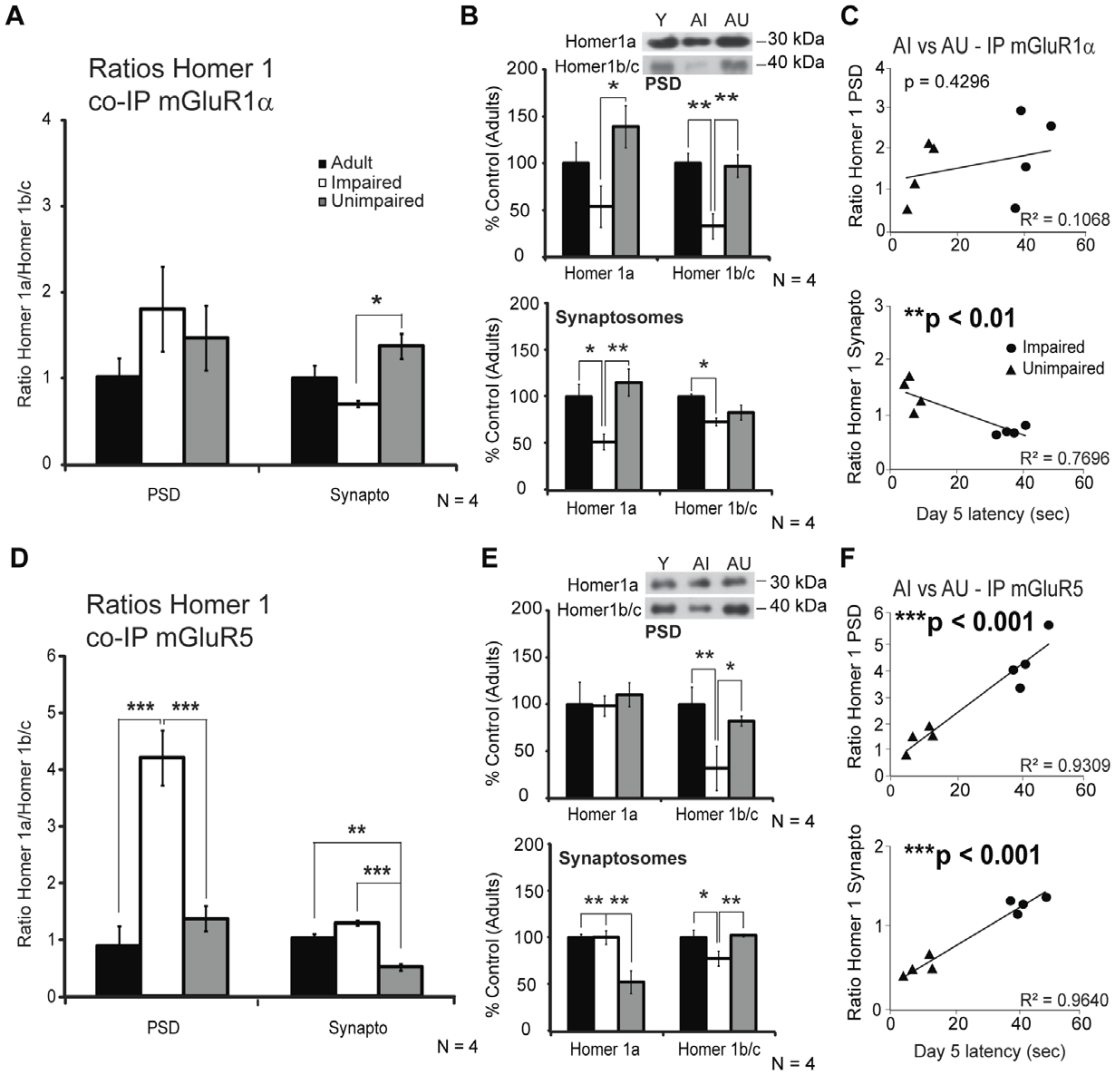
Comparison of Homer 1 isoforms ratio in adult (Y), AI and AU hippocampal formation. A) mGluR1α receptors were immunoprecipitated for PSD and synaptosomes preparations and Homer protein levels were determined by Western Blot. AU showed an increase in Homer 1a/Homer 1 b/c ratio but only in synaptosomes. B) Graphs for average Homer 1 protein levels bound to mGluR1α in the PSD and synaptosomes of each group. C) The aged rats MWM day 5 performances were correlated with the ratio of Homer 1 isoforms and mGluR1α in synaptosomes. D) The ratio of Homer 1 isoform protein levels co-immunoprecipitated with mGluR5 receptors was significantly greater in the PSD of AI rats. Conversely, this ratio was decreased in synaptosomes of AU animals. E) Graphs for average Homer 1 protein levels bound to mGluR5 in the PSD and synaptosomes of each group. F) The Homer 1 isoform ratio co-immunoprecipitated with mGluR5 receptors was strongly correlated with aged rats MWM performances. Data represent mean ± SEM of three separate experiments (N = 4) and are expressed as percentage of control (6-months adult rats), *p<0.05, **p<0.01, ***p<0.001.

Homer interactions have previously been shown to regulate mGluR dependent synaptic plasticity [35]. Moreover, Van Keuren-Jensen & Cline [36] demonstrated the importance of the Homer 1a/Homer 1b/c ratio for mGluR-mediated plasticity in optic tectal cells, where Homer 1a acts as a dominant negative modulator. We observed an increase in the Homer 1 ratio for mGluR1α in synaptosomes but no significant change at the PSD level in AU animals compared to AI rats (Fig. 7A, 1.38±0.38 vs 0.71±0.04, *p<0.05). Indeed in the PSD, both Homer 1a and Homer 1b/c protein levels bound to mGluR1α were significantly decreased with memory impairments resulting in a similar ratio (Fig. 7B, top graph, *p<0.05 and **p<0.01 respectively). The Homer 1 ratio in synaptosomes was significantly correlated with MWM performance (Fig. 7C, **p<0.01). Following mGluR5 immunoprecipitation in the PSD, we observed a change in the Homer 1a/Homer 1b/c ratio for AI animals (Fig. 7D, 4.21±0.48, ***p<0.001). Indeed, a reduced amount of Homer 1b/c proteins was bound to mGluR5 receptors for this group (Fig. 7E, top graph, 32%±24%, *p<0.05, **p<0.01), whereas the level remained the same for Homer 1a (99%±11%) compared to adult and AU rats. The ratio in PSD for the AU group (1.37±0.22) was higher but not significantly different from the adult animals (Fig. 7D, 0.89±0.35). However it was considerably decreased in synaptosomes compared to both adult and AI rats (Fig. 7D, 0.51±0.06, **p<0.01, ***p<0.001). The MWM performance correlates strongly with Homer 1a/Homer 1b/c proteins ratio bound to mGluR5 receptors in both PSD (Fig. 7F, ***p<0.001) and synaptosomes (***p<0.001).

### Signaling machinery and immediate early gene expression is more efficient in the hippocampal formation of aged memory-unimpaired rats

**Figure 8:**
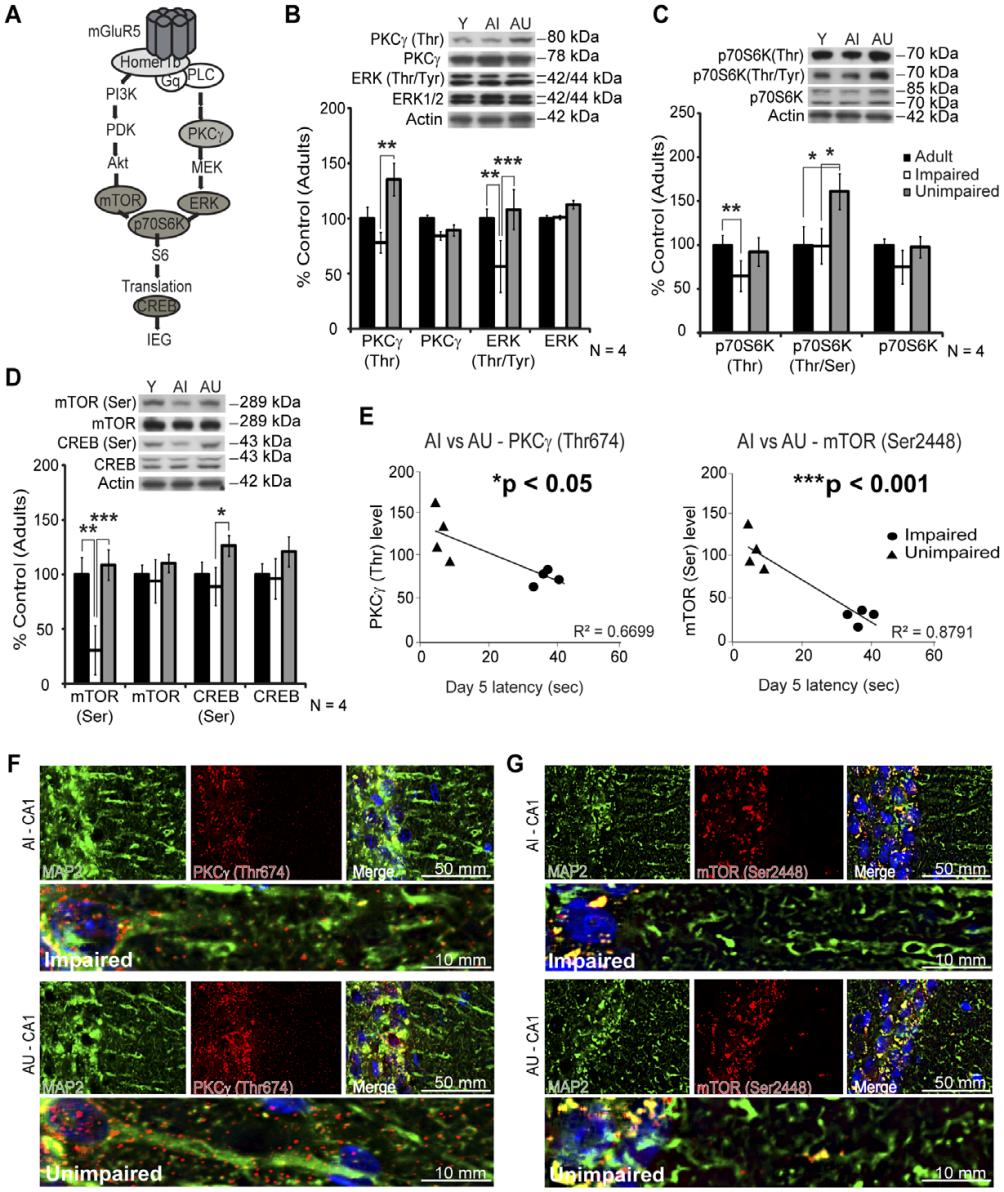
Activation of signaling pathways related to mGluR5 in adult (Y), AI and AU animals. A) Representative sketch of the multiple enzymes involved in mGluR5 activation. B) PKCγ Thr674 phosphorylation level is higher in AU homogenates while ERK 1/2 phosphorylation was decreased in AI animals. Total protein levels were unaffected in all groups. C) Thr421/Ser424 sites of p70S6 kinase enzyme were significantly more phosphorylated in AU homogenates. Total protein level remained constant in all groups. D) The strongest variation of phosphorylation level was observed for mTOR (Ser2448) in the AI group. A slight increase of CREB (Ser133) phosphorylation was also detected in AU homogenates. E) PKCγ (Thr674) and mTOR (Ser2448) phosphorylation levels were correlated with the MWM day 5 latencies in aged rats. As shown in Fig. 6F the expression of PKCγ (Thr674) was higher in dendrites of the CA1 sub-field in AU rats using double immunofluorescence with the neuronal marker MAP2. G) For mTOR (Ser2448), the increase was mostly localized in the CA1 cell bodies for AU rats. Western Blot results are represented as mean ± SEM of three separate experiments (N = 4) and the data are expressed as percentage of control (6-months adult rats), *p<0.05, **p<0.01, ***p<0.001.

Homer 1a uncouples mGluR5 from the translation machinery [35], [37] and many enzymes are associated with group 1 mGluRs receptor activation [38], [39], [40], [41]. Since alterations in signaling pathways underlying learning processes could explain memory impairments, next we evaluated the activation and protein levels of several enzymes (Fig. 8A). Protein kinase C gamma (PKCγ) is specific to neurons and colocalized with mGluR5 [42]. It has also been previously associated with learning and memory processes [43], [44], [45], [46]. PKCγ total protein level was similar in all groups (Fig. 8B); however the phosphorylation of threonine 674 residue was significantly increased in AU animals (Fig. 8B, 135%±15% vs 78%±9% for AI, **p<0.01). Extracellular signal-regulated kinase 1/2 (ERK1/2) activation and protein level were also evaluated [38], [40], [41]. ERK1/2 phosphorylation (Thr202/Tyr204) was significantly lower while the protein level remained unchanged for AI rats (Fig. 8B, 56%±24%, **p<0.01, ***p<0.001). Group 1 mGluRs activation has been previously coupled to the translation regulatory factor p70S6 kinase (p70S6K) via ERK1/2 and the mammalian target of rapamycin (mTOR) [40]. The phosphorylation of threonine 389 residue was lower in AI animals compared to adult rats but not significantly different from AU rats (Fig. 8C, 65%±18% vs 100%±11%, *p<0.05 and 92%±16%). Conversely, Thr421/Ser424 phosphorylation was specifically enhanced in the AU animals (Fig. 8C, 161%±20%, *p<0.05) but total protein levels remained unchanged. A dramatic decrease in mTOR phosphorylation (Ser2448) was observed for the AI group (Fig. 8D, 31%±22%, **p<0.01, ***p<0.001) and alteration in mTOR translational control has been previously associated with learning and memory deficits [47]. Finally, the inhibition of c-AMP response element binding protein (CREB) has been shown to disrupt mice reactivated spatial memory [48]. An increase in CREB Ser133 phosphorylation with no change in CREB expression was observed in the AU group (Fig. 8D, 126%±9%, *p<0.05). The activation of signaling enzymes PKCγ and mTOR was significantly correlated with MWM day 5 latencies (Fig. 8E, *p<0.05, ***p<0.001, respectively). Using double immunofluorescence with the neuron-specific microtubule-associated protein 2 (MAP2), we confirmed the higher level of PKCγ activation in AU hippocampus. As shown in Figure 8F, PKCγ (Thr674) level is increased in the neurons of the CA1 sub-field for AU animals, particularly in the dendrites. Unsurprisingly, mTOR (Ser2448) is present close to the nuclei and again more abundant in CA1 neurons of the AU animals (Fig. 8G). Only a few proteins were observed in the dendrites of AU animals.

**Figure 9:**
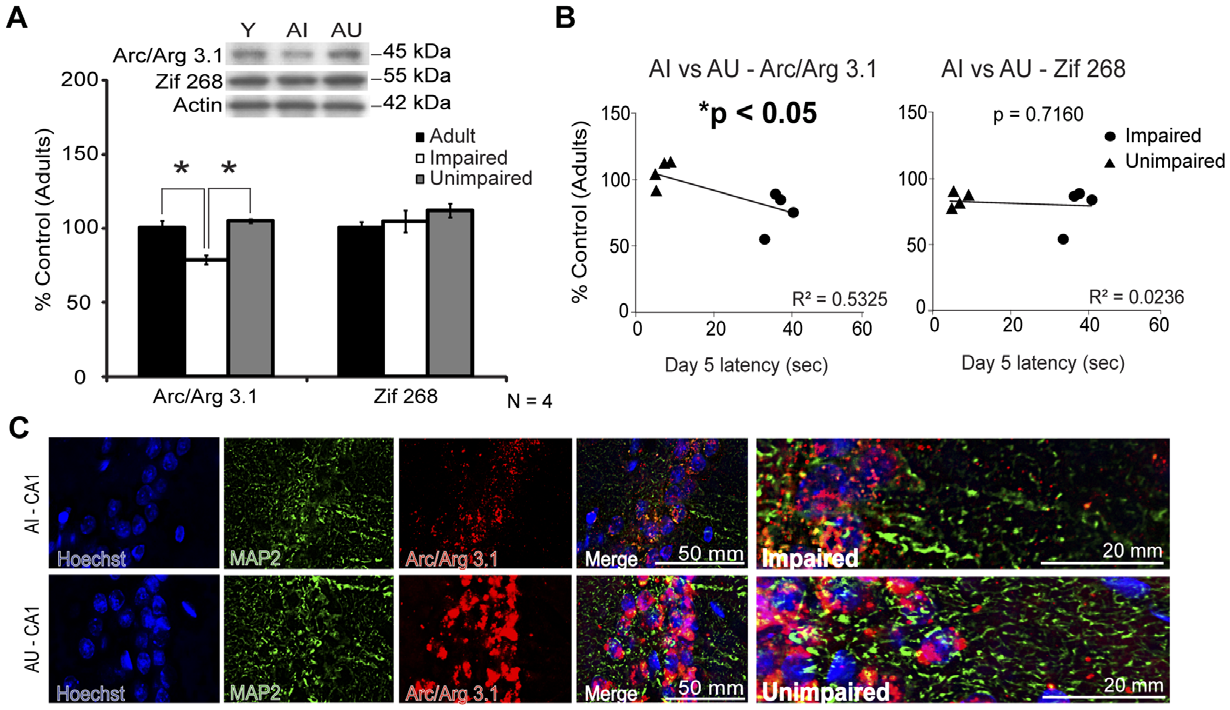
IEG Arc/Arg 3.1 and Zif 268 levels in adult (Y), AI and AU hippocampal homogenates. A) Arc/Arg 3.1 level was significantly decreased in AI homogenates while Zif 268 was unaltered. B) Arc/Arg 3.1 expression level was correlated to aged rats MWM performance. C) Arc staining was higher for the AU animals in the CA1 sub-field nuclei. Western Blot values represent mean ± SEM of three separate experiments (N = 4) and the data are expressed as percentage of control (6-months adult rats), *p<0.05.

Transcription of the IEG Arc/Arg 3.1 modulates LTP consolidation, LTD and homeostatic plasticity in the hippocampus [49]. Considering the lower activation of mGluR5 signaling pathways in the AI rat hippocampal formation following the MWM training, Arc/Arg 3.1 expression and localization was investigated. As expected, Arc/Arg 3.1 level was decreased in AI animals (Fig. 9A, 79%±3%, *p<0.05) compared to AU and young adult rats. This change was correlated with the MWM performance (Fig. 9B, *p<0.05). Another IEG, Zif 268 is important for synaptic plasticity [50] and the expression of LTP and long-term memory [51]. No significant change was observed for this IEG (Fig. 9A and Fig. 9B). Higher Arc/Arg 3.1 level for AU compared to AI animals was confirmed by double immunofluorescence with MAP2 (Fig. 9C). Most of the proteins were detected in the nuclei of the CA1 region neuronal cells.

## Discussion

In the present study, we report for the first time an involvement of mGluR5 receptors, Homer 1 proteins, and their downstream signaling pathways in hippocampus-dependent spatial memory for successful cognitive aging. Using the MWM, two distinct groups of aged animals were characterized by different learning abilities in a Long Evans rat population. Following the MWM task, we observed an up-regulation of mGluR5 protein level in homogenates and PSD of AU compared to the AI group, while mGluR1α levels were altered only in the PSD. These changes were significantly correlated with MWM performances of day 5. Interestingly, the scaffolding proteins, Homer 1a and Homer 1b/c were increased in PSD of AU animals. However, a lower ratio of Homer 1a/1b/c proteins was co-immunoprecipitated with mGluR5 receptors in the AU group, suggesting strengthened signaling pathways. This hypothesis was confirmed by Western Blot analysis of PKCγ (Thr674), ERK 1/2 (Thr202/Tyr204), p70S6K (Thr421/Ser424), mTOR (Ser2448) and CREB (Ser133). Higher PKCγ (Thr674) and mTOR (Ser2448) levels in AU hippocampus CA1 sub-field were confirmed in the dendrites and cell bodies, respectively, by double immunofluorescence with the neuronal marker MAP2. Memory consolidation is associated with the expression of IEG and a lower Arc level observed in the AI group, suggests an altered synaptic plasticity. Hence, mGluR5 and Homer 1 proteins expressed in the PSD may play a key role in the regulation of IEG.

LTD associated with Group 1 mGluR has been previously related to memory processing in Long Evans rats. While NMDA-dependent LTD was correlated with MWM performance in adult rats, NMDA-independent LTD was linked to successful aging [13]. In the present study, we modified the protocol to assess not only acquisition of spatial memory but also inhibitory learning (reverse memory) and retention (remote memory). The average latency during the reverse memory task was minimally improved in AI animals, suggesting a severe impairment to encode new spatial information. In agreement with this result, Xu et al. [31] observed significant deficits in MWM reverse memory task in mGluR5 knockout mice and proposed a role of this receptor in inhibitory learning. Increased level of mGluR5 receptor protein in the PSD following MWM training was not observed in AI and adult rats, highlighting the critical role of this receptor in normal cognition during aging. A decrease in mGluRs-mediated phosphoinositide signal transduction has already been reported in AI animals without significant changes in mGluR5 receptor protein [52]. Injection of pharmacological agents related to mGluR5 activity could be an intriguing future set of experiments to confirm or infirm the conclusions presented here. Unfortunately the survival rate of Long Evans rat population decreases exponentially with age and reaches to only 12% at 24 months (mean of 3 cohorts of 300 rats each at 12 months of age), making pharmacological studies in this animal model very difficult and expensive to pursue.

Other rat strains also present alterations in cognitive abilities with aging. While 20–40% of Long Evans rats become memory-impaired at 24–26 months of age in the MWM task, 45–100% of Fisher rats population (depending on the study) are classified as memory-impaired at the same age [5]. Sprague Dawley rats also show memory impairments in short- and long-term memory recognition tasks at 24 months [53]. Age-related decrease of NMDA-mediated synaptic plasticity has been associated with such impairments [53]. mGluR5 and Homer 1 protein levels have never been investigated in these animal models after learning/training. Thus this could be of a great interest to confirm our data in the Long Evans rat model.

**Figure 10:**
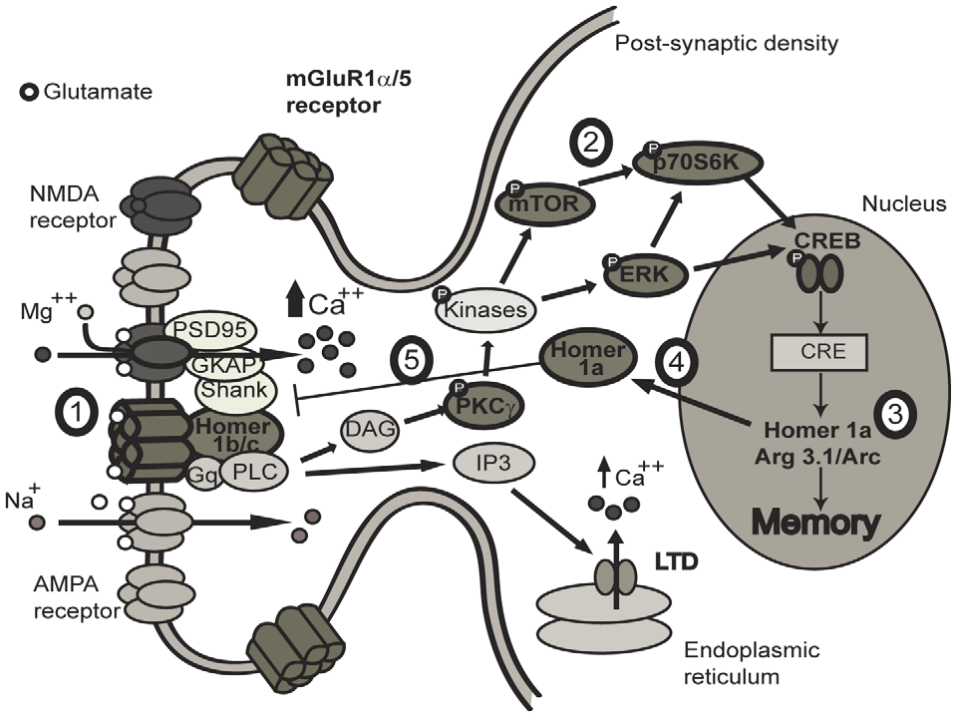
Possible mechanisms activated in the aged memory-unimpaired PSD following MWM training. MWM training induces release of glutamate which then activates group 1 mGluRs on the plasma membrane (1). Metabotropic mGluR5 are linked to ionotropic glutamate receptors by multiple scaffolding proteins (Homer 1 b/c, Shank, GKAP, PSD95). Following mGluR activation, protein kinases PKCγ, ERK1/2, mTOR, p70S6K and CREB become phosphorylated (2) and induce IEG expression (3). Homer 1a is released in the cytosol and translocated to plasma membrane (4) uncoupling the receptors with its signaling effectors (5). High levels of mGluR5 receptors bound to Homer long isoforms in the PSD of the AU rats maintain the activation of crucial signaling pathways and IEG expression following spatial memory training.

Homer proteins act as scaffolding support for the group 1 metabotropic glutamate receptors [24], [25]. Since AU animals demonstrated higher levels of mGluR5 receptors, increase in the associated Homer proteins was expected. Both Homer 1a and 1b/c levels were increased in the PSD of those animals. However the ratio of Homer 1a/Homer 1b/c bound to mGluR5 was higher in the PSD of AI group. Based on the results obtained in this study for Long Evans AU rats, here we propose a model (Fig. 10) to summarize the mechanisms implicated in successful aging after learning/training. First, a basal high level of mGluR5 binding to the long Homer protein isoforms in the PSD is required. Following MWM training, mGluR5-related signaling pathways are activated and PKCγ is phosphorylated. The role of this enzymatic activity has been reported in learning and spatial memory [46] and the levels of PKCγ increase with age [43]. In the present study, an increase in the phosphorylation of PKCγ (Thr674) was seen only in AU animals whereas ERK1/2 (Thr202/Tyr204) and mTOR (Ser2448) phosphorylation levels were similar in both AU and young adult rats. The phosphorylation level of ERK1/2 was previously correlated to cognitive impairments related to aging [54]. Moreover, ERK plays a key role in the hippocampal CA1/CA2 regions in long-term spatial memory following multiple training trials in the MWM [55]. While AU animals optimally use mGluR5-related pathways, younger animals might favour NMDA receptors-mediated signaling. Indeed, Gong et al. [56] suggested a critical role for NMDA receptors and mTOR to induce dendritic protein synthesis in hippocampal neurons. Furthermore, spatial memory performance strongly correlates with the magnitude of NMDA-dependent LTP in young rats while NMDA-independent LTP was closely associated to cognition in aged rats [57].

Alterations in the level and phosphorylation of CREB have been reported in cognitively-impaired aged rats [58], [59]. Following MWM training, AU rats displayed high levels of phosphorylated CREB, suggesting an activation of the transcription machinery, which was confirmed by a higher level of IEG Arc especially in the CA1 area of the hippocampus. Zif268 levels were not significantly different among the groups. Since, this IEG seems particularly important for plasticity underlying the first training days [50]; it may not be relevant after three weeks. Synaptic activity also induced the synthesis of Homer 1a [23] but its sustained expression in the dorsal hippocampus of transgenic mice impairs LTP and spatial working memory [60]. Interestingly, recombinant adeno-associated virus-mediated overexpression of Homer 1a impairs hippocampal-dependent memory while the overexpression of Homer 1c slightly facilitates memory performance [61]. Thus perturbations of synaptic group 1 mGluR equilibrium by Homer 1 isoforms may occur in AI animals. Indeed these receptors could more easily be uncoupled from its downstream signaling pathways, leading to long-term lower protein synthesis required for synaptic plasticity.

Metabotropic mGluR5 receptors dynamically regulate NMDA receptor function through Homer-Shank multi-protein scaffolding and this interaction can be disrupted by Homer 1a [62]. On the other hand, the stimulation of NMDA receptors induces a positive feedback regulation of mGluR1α via calpain-mediated truncation [63]. Blocking NMDA-dependent LTD with specific NR2B antagonists impairs spatial memory consolidation [64]. Therefore, it would be of interest to evaluate possible interactions between metabotropic and ionotropic glutamate receptors in our model. Another intriguing hypothesis involving ionotropic glutamate receptors is the homeostatic scaling of the sensitivity of hippocampal synapses by group 1 mGluR and Homer 1a [65]. This form of synaptic plasticity adjusts the strength of excitatory synapses to stabilize firing [66]. Network activity induces transient Homer 1a expression and decreases the level of synaptic AMPA receptors [65]. Moreover, synapse-specific homeostatic plasticity is also observed in mature synapses and requires IEG Arc [67]. Aging likely affect these refined synaptic processes [68] and consequently information storage in the brain.

Most of the changes observed in the AI rat brains were in the CA1 hippocampal region in accordance with its role in the organization of recent memories [69]. Gene expression profiles have been investigated in the CA1, CA3 and DG areas in adult, AI and AU groups [5], [70]. Interestingly, changes observed in the CA3 region were better correlated to memory impairments [70]. However, changes in gene expression associated to lysosome, immune function and mRNA translation, were also correlated to cognitive deficits without being specific to the CA3 region [70]. Our group previously reported a reduction in the expression level of various genes in the dorsal hippocampus following scopolamine-induced amnesia including Homer 1a, GABA B receptor and Zif268 [71]. Finally, normal aging seems to affect the pre-synaptic modulation of glutamatergic synapses. Interestingly, Zhang et al. [72] reported a decrease in extracellular glutamate levels in the hippocampus of 24-months old cognitively-impaired Fischer-344 rats. It would thus be of interest to compare the integrity of pre-synaptic components in AI and AU Long Evans rats.

Excessive protein synthesis downstream mGluR5 activation has been proposed to explain the fragile X syndrome, a genetic syndrome characterized by multiple symptoms including intellectual dysfunction, social anxiety and working memory deficits. Many symptoms of this disease can be reduced by modulating mGluR5 in animal models [73], [74]. For example, the acute blockade of mGluR5 and ERK1/2 decreased protein synthesis to wild-type level in a fragile X mental retardation protein (FMRP) knockout mice model [75]. FMRP is a cytoplasmic mRNA binding protein able to regulate the translation of the amyloid precursor protein through mGluR [76]. Interestingly, the accumulation of beta-amyloid (Aβ) at synapses observed in Alzheimer's brain reduces mGluR5 lateral diffusion in the membrane leading to an aberrant accumulation of glutamate receptors, elevated intracellular Ca+ levels and the synapse dysfunction [77]. The clustering and lateral mobility of mGluRs are influenced by Homer interactions [78] and Aβ has been reported to decrease synaptic levels of Homer 1b [79]. In this perspective, it could be attractive to investigate mGluR and Homer 1 protein levels and their interactions with aging in pathological mouse models.

In this study we identified for the first time, mGluR5 glutamate receptors, Homer 1 proteins and their downstream signalling pathways as mediators of successful cognitive aging in a population of Long Evans rats. After training, more changes were observed in the hippocampal formation of aged memory-unimpaired compared to memory-impaired rats, suggesting a better adaptation of their brain plasticity to aging. Some of those variations were not observed in the 6-months adult animals, indicating a specific mechanism underlying spatial learning and memory in the aged hippocampus. Hopefully, our work will help to understand the individual differences observed with aging not only in rodents but also in humans and provide novel hypothesis to understand pathological aging.

## Supporting Information

Figure S1 **Control experiments for the specificity of mGluR5 and Homer 1 antibodies and PSD purification.** A) mGluR5 antibody strongly reacted to one specific band (132 kDa). Immunostaining was blocked by peptide preadsorption in both Western Blot and immunohistochemistry. Specificity of the anti-rabbit secondary antibody was assessed by primary antibody omission. B) Homer 1a primary antibody shows several nonspecific bands. One band between 30 and 36 kDa was blocked by pre-incubation with peptide. Specificity of the anti-goat secondary antibody was assessed by primary antibody omission. C) Homer 1b/c antibody was highly specific with only one immunoreactive band. No staining was observed following preadsorption with the peptide or when the primary antibody was omitted. D) Purification of the PSD by subcellular fractionation and Triton X-100 treatment was assessed by Western Blot using PSD95 as a marker. Actin was used as a loading control.
(TIF)

## References

[1] Rowe JW, Kahn RL (1997). Successful aging.. The Gerontologist.

[2] Aubert I, Rowe W, Meaney MJ, Gauthier S, Quirion R (1995). Cholinergic markers in aged cognitively impaired Long-Evans rats.. Neuroscience.

[3] Rowe WB, Spreekmeester E, Meaney MJ, Quirion R, Rochford J (1998). Reactivity to novelty in cognitively-impaired and cognitively-unimpaired aged rats and young rats.. Neuroscience.

[4] Gallagher M, Bizon JL, Hoyt EC, Helm KA, Lund PK (2003). Effects of aging on the hippocampal formation in a naturally occurring animal model of mild cognitive impairment.. Experimental gerontology.

[5] Benoit CE, Rowe WB, Menard C, Sarret P, Quirion R (2011). Genomic and proteomic strategies to identify novel targets potentially involved in learning and memory.. Trends in pharmacological sciences.

[6] Brouillette J, Quirion R (2008). Transthyretin: a key gene involved in the maintenance of memory capacities during aging.. Neurobiology of aging.

[7] Rowe WB, Blalock EM, Chen KC, Kadish I, Wang D (2007). Hippocampal expression analyses reveal selective association of immediate-early, neuroenergetic, and myelinogenic pathways with cognitive impairment in aged rats.. The Journal of neuroscience: the official journal of the Society for Neuroscience.

[8] Wilson IA, Gallagher M, Eichenbaum H, Tanila H (2006). Neurocognitive aging: prior memories hinder new hippocampal encoding.. Trends in neurosciences.

[9] Bredt DS, Nicoll RA (2003). AMPA receptor trafficking at excitatory synapses.. Neuron.

[10] Lee YS, Silva AJ (2009). The molecular and cellular biology of enhanced cognition.. Nature reviews Neuroscience.

[11] Lamprecht R, LeDoux J (2004). Structural plasticity and memory.. Nature reviews Neuroscience.

[12] Barnes CA (2003). Long-term potentiation and the ageing brain.. Philosophical transactions of the Royal Society of London Series B, Biological sciences.

[13] Lee HK, Min SS, Gallagher M, Kirkwood A (2005). NMDA receptor-independent long-term depression correlates with successful aging in rats.. Nature neuroscience.

[14] Palmer MJ, Irving AJ, Seabrook GR, Jane DE, Collingridge GL (1997). The group I mGlu receptor agonist DHPG induces a novel form of LTD in the CA1 region of the hippocampus.. Neuropharmacology.

[15] Nicoletti F, Bockaert J, Collingridge GL, Conn PJ, Ferraguti F (2011). Metabotropic glutamate receptors: from the workbench to the bedside.. Neuropharmacology.

[16] Collett VJ, Collingridge GL (2004). Interactions between NMDA receptors and mGlu5 receptors expressed in HEK293 cells.. British journal of pharmacology.

[17] Romano C, van den Pol AN, O'Malley KL (1996). Enhanced early developmental expression of the metabotropic glutamate receptor mGluR5 in rat brain: protein, mRNA splice variants, and regional distribution.. The Journal of comparative neurology.

[18] Bortolotto ZA, Collett VJ, Conquet F, Jia Z, van der Putten H (2005). The regulation of hippocampal LTP by the molecular switch, a form of metaplasticity, requires mGlu5 receptors.. Neuropharmacology.

[19] Manahan-Vaughan D, Braunewell KH (2005). The metabotropic glutamate receptor, mGluR5, is a key determinant of good and bad spatial learning performance and hippocampal synaptic plasticity.. Cerebral cortex.

[20] Bikbaev A, Neyman S, Ngomba RT, Conn PJ, Nicoletti F (2008). MGluR5 mediates the interaction between late-LTP, network activity, and learning.. PloS one.

[21] Neyman S, Manahan-Vaughan D (2008). Metabotropic glutamate receptor 1 (mGluR1) and 5 (mGluR5) regulate late phases of LTP and LTD in the hippocampal CA1 region in vitro.. The European journal of neuroscience.

[22] Bird CM, Burgess N (2008). The hippocampus and memory: insights from spatial processing.. Nature reviews Neuroscience.

[23] Brakeman PR, Lanahan AA, O'Brien R, Roche K, Barnes CA (1997). Homer: a protein that selectively binds metabotropic glutamate receptors.. Nature.

[24] Xiao B, Tu JC, Petralia RS, Yuan JP, Doan A (1998). Homer regulates the association of group 1 metabotropic glutamate receptors with multivalent complexes of homer-related, synaptic proteins.. Neuron.

[25] Xiao B, Tu JC, Worley PF (2000). Homer: a link between neural activity and glutamate receptor function.. Current opinion in neurobiology.

[26] Fagni L, Worley PF, Ango F (2002). Homer as both a scaffold and transduction molecule.. Science's STKE: signal transduction knowledge environment.

[27] Yang L, Mao L, Tang Q, Samdani S, Liu Z (2004). A novel Ca2+-independent signaling pathway to extracellular signal-regulated protein kinase by coactivation of NMDA receptors and metabotropic glutamate receptor 5 in neurons.. The Journal of neuroscience: the official journal of the Society for Neuroscience.

[28] Duncan RS, Hwang SY, Koulen P (2005). Effects of Vesl/Homer proteins on intracellular signaling.. Experimental biology and medicine.

[29] Mao L, Yang L, Tang Q, Samdani S, Zhang G (2005). The scaffold protein Homer1b/c links metabotropic glutamate receptor 5 to extracellular signal-regulated protein kinase cascades in neurons.. The Journal of neuroscience: the official journal of the Society for Neuroscience.

[30] Morris R (1984). Developments of a water-maze procedure for studying spatial learning in the rat.. Journal of neuroscience methods.

[31] Xu J, Zhu Y, Contractor A, Heinemann SF (2009). mGluR5 has a critical role in inhibitory learning.. The Journal of neuroscience: the official journal of the Society for Neuroscience.

[32] Lauterborn JC, Lynch G, Vanderklish P, Arai A, Gall CM (2000). Positive modulation of AMPA receptors increases neurotrophin expression by hippocampal and cortical neurons.. The Journal of neuroscience: the official journal of the Society for Neuroscience.

[33] Henley JM (1995). Subcellular localization and molecular pharmacology of distinct populations of [3H]-AMPA binding sites in rat hippocampus.. British Journal of Pharmacology.

[34] Moron JA, Abul-Husn NS, Rozenfeld R, Dolios G, Wang R (2007). Morphine administration alters the profile of hippocampal postsynaptic density-associated proteins: a proteomics study focusing on endocytic proteins.. Molecular & cellular proteomics: MCP.

[35] Kammermeier PJ, Worley PF (2007). Homer 1a uncouples metabotropic glutamate receptor 5 from postsynaptic effectors.. Proceedings of the National Academy of Sciences of the United States of America.

[36] Van Keuren-Jensen K, Cline HT (2006). Visual experience regulates metabotropic glutamate receptor-mediated plasticity of AMPA receptor synaptic transmission by homer1a induction.. The Journal of neuroscience: the official journal of the Society for Neuroscience.

[37] Ronesi JA, Huber KM (2008). Homer interactions are necessary for metabotropic glutamate receptor-induced long-term depression and translational activation.. The Journal of neuroscience: the official journal of the Society for Neuroscience.

[38] Gallagher SM, Daly CA, Bear MF, Huber KM (2004). Extracellular signal-regulated protein kinase activation is required for metabotropic glutamate receptor-dependent long-term depression in hippocampal area CA1.. The Journal of neuroscience: the official journal of the Society for Neuroscience.

[39] Hou L, Klann E (2004). Activation of the phosphoinositide 3-kinase-Akt-mammalian target of rapamycin signaling pathway is required for metabotropic glutamate receptor-dependent long-term depression.. The Journal of neuroscience: the official journal of the Society for Neuroscience.

[40] Page G, Khidir FA, Pain S, Barrier L, Fauconneau B (2006). Group I metabotropic glutamate receptors activate the p70S6 kinase via both mammalian target of rapamycin (mTOR) and extracellular signal-regulated kinase (ERK 1/2) signaling pathways in rat striatal and hippocampal synaptoneurosomes.. Neurochemistry international.

[41] Antion MD, Hou L, Wong H, Hoeffer CA, Klann E (2008). mGluR-dependent long-term depression is associated with increased phosphorylation of S6 and synthesis of elongation factor 1A but remains expressed in S6K-deficient mice.. Molecular and cellular biology.

[42] Liu JX, Tang YC, Liu Y, Tang FR (2008). mGluR5-PLCbeta4-PKCbeta2/PKCgamma pathways in hippocampal CA1 pyramidal neurons in pilocarpine model of status epilepticus in mGluR5+/+ mice.. Epilepsy research.

[43] Colombo PJ, Wetsel WC, Gallagher M (1997). Spatial memory is related to hippocampal subcellular concentrations of calcium-dependent protein kinase C isoforms in young and aged rats.. Proceedings of the National Academy of Sciences of the United States of America.

[44] Colombo PJ, Gallagher M (2002). Individual differences in spatial memory among aged rats are related to hippocampal PKCgamma immunoreactivity.. Hippocampus.

[45] Saito N, Shirai Y (2002). Protein kinase C gamma (PKC gamma): function of neuron specific isotype.. Journal of biochemistry.

[46] Nithianantharajah J, Murphy M (2009). Experience on the Barnes spatial maze influences PKCgamma levels in the hippocampus.. The International journal of neuroscience.

[47] Parsons RG, Gafford GM, Helmstetter FJ (2006). Translational control via the mammalian target of rapamycin pathway is critical for the formation and stability of long-term fear memory in amygdala neurons.. The Journal of neuroscience: the official journal of the Society for Neuroscience.

[48] Kim R, Moki R, Kida S (2011). Molecular mechanisms for the destabilization and restabilization of reactivated spatial memory in the Morris water maze.. Molecular brain.

[49] Bramham CR, Worley PF, Moore MJ, Guzowski JF (2008). The immediate early gene arc/arg3.1: regulation, mechanisms, and function.. The Journal of neuroscience: the official journal of the Society for Neuroscience.

[50] Alberini CM (2009). Transcription factors in long-term memory and synaptic plasticity.. Physiological reviews.

[51] Jones MW, Errington ML, French PJ, Fine A, Bliss TV (2001). A requirement for the immediate early gene Zif268 in the expression of late LTP and long-term memories.. Nature neuroscience.

[52] Nicolle MM, Colombo PJ, Gallagher M, McKinney M (1999). Metabotropic glutamate receptor-mediated hippocampal phosphoinositide turnover is blunted in spatial learning-impaired aged rats.. The Journal of neuroscience: the official journal of the Society for Neuroscience.

[53] Kollen M, Stephan A, Faivre-Bauman A, Loudes C, Sinet PM (2010). Preserved memory capacities in aged Lou/C/Jall rats.. Neurobiology of aging.

[54] Williams BJ, Bimonte-Nelson HA, Granholm-Bentley AC (2006). ERK-mediated NGF signaling in the rat septo-hippocampal pathway diminishes with age.. Psychopharmacology.

[55] Blum S, Moore AN, Adams F, Dash PK (1999). A mitogen-activated protein kinase cascade in the CA1/CA2 subfield of the dorsal hippocampus is essential for long-term spatial memory.. The Journal of neuroscience: the official journal of the Society for Neuroscience.

[56] Gong R, Park CS, Abbassi NR, Tang SJ (2006). Roles of glutamate receptors and the mammalian target of rapamycin (mTOR) signaling pathway in activity-dependent dendritic protein synthesis in hippocampal neurons.. The Journal of biological chemistry.

[57] Boric K, Munoz P, Gallagher M, Kirkwood A (2008). Potential adaptive function for altered long-term potentiation mechanisms in aging hippocampus.. The Journal of neuroscience: the official journal of the Society for Neuroscience.

[58] Brightwell JJ, Gallagher M, Colombo PJ (2004). Hippocampal CREB1 but not CREB2 is decreased in aged rats with spatial memory impairments.. Neurobiology of learning and memory.

[59] Monti B, Berteotti C, Contestabile A (2005). Dysregulation of memory-related proteins in the hippocampus of aged rats and their relation with cognitive impairment.. Hippocampus.

[60] Celikel T, Marx V, Freudenberg F, Zivkovic A, Resnik E (2007). Select overexpression of homer1a in dorsal hippocampus impairs spatial working memory.. Frontiers in neuroscience.

[61] Klugmann M, Symes CW, Leichtlein CB, Klaussner BK, Dunning J (2005). AAV-mediated hippocampal expression of short and long Homer 1 proteins differentially affect cognition and seizure activity in adult rats.. Molecular and cellular neurosciences.

[62] Bertaso F, Roussignol G, Worley P, Bockaert J, Fagni L (2010). Homer1a-dependent crosstalk between NMDA and metabotropic glutamate receptors in mouse neurons.. PloS one.

[63] Xu W, Wong TP, Chery N, Gaertner T, Wang YT (2007). Calpain-mediated mGluR1alpha truncation: a key step in excitotoxicity.. Neuron.

[64] Ge Y, Dong Z, Bagot RC, Howland JG, Phillips AG (2010). Hippocampal long-term depression is required for the consolidation of spatial memory.. Proceedings of the National Academy of Sciences of the United States of America.

[65] Hu JH, Park JM, Park S, Xiao B, Dehoff MH (2010). Homeostatic scaling requires group I mGluR activation mediated by Homer1a.. Neuron.

[66] Turrigiano GG (2008). The self-tuning neuron: synaptic scaling of excitatory synapses.. Cell.

[67] Beique JC, Na Y, Kuhl D, Worley PF, Huganir RL (2011). Arc-dependent synapse-specific homeostatic plasticity.. Proceedings of the National Academy of Sciences of the United States of America.

[68] Burke SN, Barnes CA (2010). Senescent synapses and hippocampal circuit dynamics.. Trends in neurosciences.

[69] Frankland PW, Bontempi B (2005). The organization of recent and remote memories.. Nature reviews Neuroscience.

[70] Haberman RP, Colantuoni C, Stocker AM, Schmidt AC, Pedersen JT (2011). Prominent hippocampal CA3 gene expression profile in neurocognitive aging.. Neurobiology of aging.

[71] Brouillette J, Young D, During MJ, Quirion R (2007). Hippocampal gene expression profiling reveals the possible involvement of Homer1 and GABA(B) receptors in scopolamine-induced amnesia.. Journal of neurochemistry.

[72] Zhang WQ, Mundy WR, Thai L, Hudson PM, Gallagher M (1991). Decreased glutamate release correlates with elevated dynorphin content in the hippocampus of aged rats with spatial learning deficits.. Hippocampus.

[73] Dolen G, Bear MF (2008). Role for metabotropic glutamate receptor 5 (mGluR5) in the pathogenesis of fragile X syndrome.. The Journal of physiology.

[74] Krueger DD, Bear MF (2011). Toward fulfilling the promise of molecular medicine in fragile X syndrome.. Annual review of medicine.

[75] Osterweil EK, Krueger DD, Reinhold K, Bear MF (2010). Hypersensitivity to mGluR5 and ERK1/2 leads to excessive protein synthesis in the hippocampus of a mouse model of fragile X syndrome.. The Journal of neuroscience: the official journal of the Society for Neuroscience.

[76] Westmark CJ, Malter JS (2007). FMRP mediates mGluR5-dependent translation of amyloid precursor protein.. PLoS biology.

[77] Renner M, Lacor PN, Velasco PT, Xu J, Contractor A (2010). Deleterious effects of amyloid beta oligomers acting as an extracellular scaffold for mGluR5.. Neuron.

[78] Serge A, Fourgeaud L, Hemar A, Choquet D (2002). Receptor activation and homer differentially control the lateral mobility of metabotropic glutamate receptor 5 in the neuronal membrane.. The Journal of neuroscience: the official journal of the Society for Neuroscience.

[79] Roselli F, Hutzler P, Wegerich Y, Livrea P, Almeida OF (2009). Disassembly of shank and homer synaptic clusters is driven by soluble beta-amyloid(1–40) through divergent NMDAR-dependent signalling pathways.. PloS one.

