# Supplementary figures and images for: Successful Cognitive Aging in Rats: A Role for mGluR5 Glutamate Receptors, Homer 1 Proteins and Downstream Signaling Pathways
Source: PLoS One. 2012 Jan 6;7(1):e28666. doi: 10.1371/journal.pone.0028666 (PMC3253083; doi:10.1371/journal.pone.0028666)

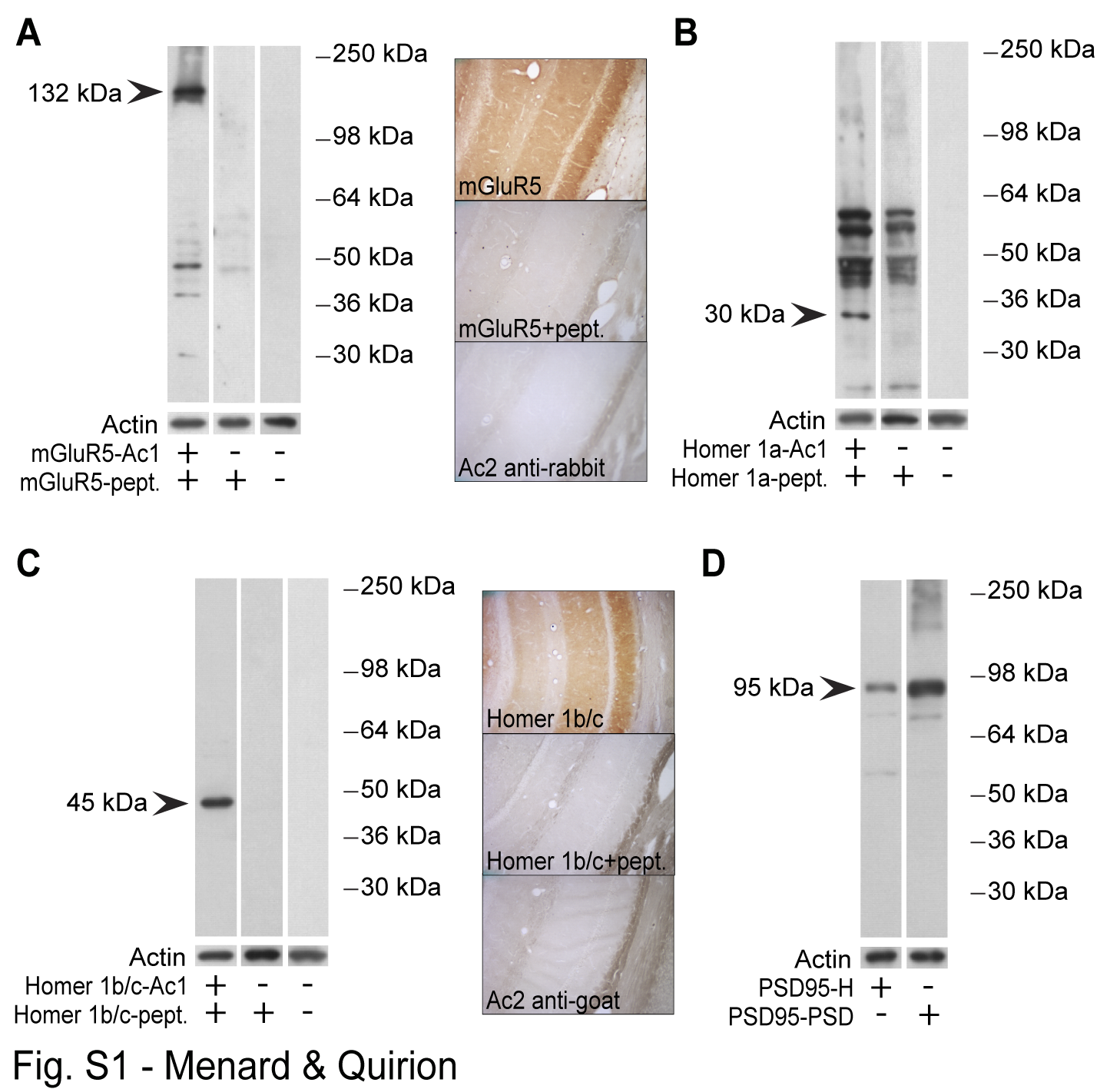

Supplement: Figure S1 — Control experiments for the specificity of mGluR5 and Homer 1 antibodies and PSD purification. A) mGluR5 antibody strongly reacted to one specific band (132 kDa). Immunostaining was blocked by peptide preadsorption in both Western Blot and immunohistochemistry. Specificity of the anti-rabbit secondary antibody was assessed by primary antibody omission. B) Homer 1a primary antibody shows several nonspecific bands. One band between 30 and 36 kDa was blocked by pre-incubation with peptide. Specificity of the anti-goat secondary antibody was assessed by primary antibody omission. C) Homer 1b/c antibody was highly specific with only one immunoreactive band. No staining was observed following preadsorption with the peptide or when the primary antibody was omitted. D) Purification of the PSD by subcellular fractionation and Triton X-100 treatment was assessed by Western Blot using PSD95 as a marker. Actin was used as a loading control. (TIF) [file pone.0028666.s001.tif]
